# Staffing in public health facilities after the Ebola outbreak in rural Sierra Leone: How much has changed?

**DOI:** 10.12688/f1000research.18566.2

**Published:** 2020-01-09

**Authors:** James Sylvester Squire, Katrina Hann, Olga Denisiuk, Rony Zachariah

**Affiliations:** 1 Directorate of Health Security and Emergencies, Ministry of Health and Sanitation, Freetown, Sierra Leone; 2 Sustainable Health Systems, Freetown, Sierra Leone; 3 Alliance for Public Health, Kiev, Ukraine; 4 TDR, The Special Programme for Research and Training in Tropical Diseases (WHO/TDR), Geneva, Switzerland

**Keywords:** Outbreak response, SORT IT, Sustainable Development Goals, Universal Health Coverage, Basic Package of Essential Health Services

## Abstract

**Background:** The 2014-2015 Ebola outbreak in Sierra Leone led the Ministry of Health and Sanitation to set minimum standards of staffing (medical/non-medical) at the district level for the provision of basic essential health services (BPEHS).

In one of the worst Ebola affected districts in Sierra Leone, we assessed staffing levels measured against these stipulated standards before, during, and 16 months after the Ebola outbreak.

**Methods:** The study population included all health workers in 83 health facilities. We assessed staffing levels at three points in time: pre-Ebola (April 2014); the end of the outbreak (November 2015); and 16 months post-Ebola (March 2017).

April 2014 was immediately prior to the Ebola outbreak and thus representative of the human resource situation before the outbreak. November 2015 was the month when Sierra Leone was declared Ebola-free, and thus reflects the end-situation after Ebola. March 2017 was two years since the launch of the BPEHS, and some progress should be expected.

**Results:** Against recommended medical staff numbers during pre-, intra- and post-Ebola periods, deficits were 67%, 65% and 60% respectively. Similarly, against recommended non-medical staff numbers during pre-, intra- and post-Ebola periods, the deficit remained at 92% throughout. In the post-Ebola period, there was a deficit of 73% against 1,389 recommended health worker positions.

**Conclusions:** *Nothing has really changed* in the state of human resources for health, and urgent measures are needed to rectify the situation and prevent a *déjà vu* in the advent of a new Ebola outbreak.

## Introduction

The Ministry of Health and Sanitation of Sierra Leone has stipulated minimum staffing levels for all public health facility levels based on the Basic Package of Essential Health Services (BPEHS) ^1^. An observational study published in 2017 following the 2014–2015 Ebola outbreak reported alarming human resource deficits in public health facilities in Kailahun district of rural Sierra Leone ^2^. Of 805 recommended medical staff, the deficit was 501 (62%) and hovered over 50% at all levels of health facilities. Similarly, of 569 recommended non-medical staff, the deficit was 524 (92%). The overarching message was that to meet the BPEHS ^1^ standards, the Government would need to attract an additional 1,026 workers to Kailahun district over the period 2016–2020 (roughly 256 additional workers per annum).

The post-Ebola period presented an opportunity for the Government of Sierra Leone to devise a health services investment plan. This included a robust investment in human resources for health with a 2020 target for scale up of the BPEHS ^3^. Both medical and non-medical staff are essential to maintain service delivery standards, including infection prevention and control practices, and both these staff cadres are included. The shortage in non-medical staff was found to have major implications for maintaining essential services related to infection prevention and control (IPC), such as screening and triage, health facility and personal hygiene as well as waste management ^2^.

Three years have now passed since the end of the Ebola outbreak and the operational question is “what has changed” in terms of progress towards achieving BPEHS standards.

Among all public health facilities in Kailahun district of Sierra Leone and in relation to BPEHS standards, we thus assessed staffing levels (medical and non-medical) one month before the onset of the Ebola outbreak, during the last month of the outbreak, and 16 months thereafter.

## Methods

This was a comparative cross-sectional study using programme data. The study setting has been described before ^2^. The study site was Kailahun district, the first district affected by the Ebola outbreak in Sierra Leone. It shares borders with the Republic of Liberia and Guinea. The health infrastructure is tiered into tertiary hospitals, district hospitals and Peripheral Health Units. The current study included all 82 functional public health facilities.

Pre-service training of health care workers is carried out by the Ministry of Tertiary and Higher Education. Upon completion of training, health workers are recruited by the Public Service Commission of the Government of Sierra Leone. Financing of health workers once recruited into the civil service is largely carried out by the Government of Sierra Leone through a consolidated fund. The Human Resource Directorate of the Ministry of Health and Sanitation has the responsibility of posting staff to health facilities across the country. However, due to budgetary limitations on paying salaries, many health care workers serve as volunteers in health facilities and are not on a regular payroll. The International Monetary Fund macro-economic restrictions on fiscal space, in particular the wage bill, hampers the recruitment and hiring of health workers ^4^, and this has been a major barrier in increasing staff numbers in relation to the BPEHS.

The study population included all health workers in these health facilities. We disaggregated staff deficits by medical and non-medical staff (for a full list see Table 5 & Table 6 of Squire et al. 2017) ^2^. We assessed staffing levels at 16 months post-Ebola (March 2017), and compared to previously reported staffing levels for pre-Ebola (April 2014) and the end of the outbreak (November 2015) ^1^.

April 2014 was immediately prior to the Ebola outbreak and thus representative of the human resource situation before the outbreak. November 2015 was the month when Sierra Leone was declared Ebola-free, and thus representative of the end-situation after Ebola. March 2017 was selected because the revised BPEHS was launched two years prior to this date, and some progress should have been expected.

Data variables were sourced from the monthly district staff list (District Health Information Systems; DHIS2) and the Human Resource Management Information System. Deficits in staffing levels were derived by subtracting the actual levels from the stipulated levels.

Ethics approval was obtained from the Sierra Leone Ethics and Scientific Review Board (dated 18 December 2018) and the Union Ethics Advisory Group (International Union against Tuberculosis and Lung Disease, Paris, France; UAG number 71/18). Since anonymized programme data were used, the requirement for informed consent was waived.

## Results

Table 1 shows the *medical staffing* levels in relation to BPEHS standards. Of 805 recommended medical staff during the pre-Ebola and intra-Ebola periods, deficits were 539 (67%) and 528 (65%) respectively. During the post-Ebola period, a total of 815 medical staff were recommended, but the deficit was 490 (60%; a 5% improvement over the intra-Ebola period). When stratified by health facility levels, human resource gaps ranged between 31% and 71%.

**Table 1.** Overall medical staffing ^1^ levels and gaps in relation to the recommended BPEHS standards assessed pre-, intra- and post-Ebola ^2^ in Kailahun district, Sierra Leone.

		Pre-Ebola n (%)	Intra-Ebola n (%)	Post-Ebola n (%)
**Total staff**	Recommended	805	805	815 ^3^
	Actual	266	277	325
	Human resource gap	539 (67)	528 (66)	490 (60)
**Health facility levels**				
District Hospital	Recommended	256	256	256
	Actual	66	77	74
	Human resource gap	190 (74)	179 (70)	182 (71)
CHC	Recommended	252	252	252
	Actual	71	77	97
	Human resource gap	181 (72)	175 (69)	155 (62)
CHP	Recommended	240	240	265 ^4^
	Actual	104	101	125
	Human resource gap	136 (57)	139 (58)	140 (53)
MCHP	Recommended	57	57	42 ^4^
	Actual	25	22	29
	Human resource gap	32 (56)	35 (61)	13 (31)

BPEHS: Basic Package of Essential Health Services document for improving health service delivery in Sierra Leone; CHC: Community Health Center; CHP: Community Health Post; MCHP: Maternal and Child Health Post ^1^ Includes staff such as specialist doctors, general practitioners, clinical officers, nurses and midwives ^2 ^Pre-Ebola – April 2014; Intra-Ebola – November 2015; Post-Ebola – March 2017 ^3 ^The overall recommended numbers of staff as per the BPEHS increased from 805 during the pre- and intra-Ebola period to 815 in the post-Ebola period as one new facility was added in the post-Ebola period. ^4^ Similarly, during the post-Ebola period, 5 MCHPs were upgraded to CHPs increasing the staffing requirement for the CHPs from 240 to 265.

Table 2 shows *non-medical staffing* levels in relation to BPEHS standards. The overall deficit remained the same at the three time-points. Of 569 recommended non-medical staff during pre- and post-Ebola, the deficits were 526 (92%) and 525 (92%), respectively. During the post-Ebola period, of 574 recommended non-medical staff, the deficit was 528 (92%).

**Table 2.** Overall non-medical ^1^ staffing levels and gaps in relation to the recommended BPEHS standards assessed pre-, intra- and post-Ebola ^2^ in Kailahun district, Sierra Leone.

		Pre-Ebola n (%)	Intra-Ebola n (%)	Post-Ebola n (%)
**Total staff**	Recommended	569	569	574 ^3^
	Actual	43	44	46
	Human resource gap	526 (92)	525 (92)	528 (92)
**Health facility levels**				
District Hospital	Recommended	88	88	88
	Actual	31	31	34
	Human resource gap	57 (65)	57 (65)	54 (61)
CHC	Recommended	98	98	98
	Actual	9	9	8
	Human resource gap	89 (91)	89 (91)	90 (92)
CHP	Recommended	288	288	318 ^4^
	Actual	3	4	4
	Human resource gap	285 (99)	284 (99)	314 (99)
MCHP	Recommended	95	95	70 ^4^
	Actual	0	0	0
	Human resource gap	95 (100)	95 (100)	70 (100)

BPEHS: Basic Package of Essential Health Services document for improving health service delivery in Sierra Leone; CHC: Community Health Center; CHP: Community Health Post; MCHP: Maternal and Child Health Post ^1^ Includes staff such as administrative staff, cleaners, cooks, maintenance workers, drivers and security personnel ^2 ^Pre-Ebola – April 2014; Intra-Ebola – November 2015; Post-Ebola – March 2017 **** ^3 ^The overall recommended numbers of staff as per the BPEHS increased from 569 during the pre- and intra-Ebola period to 574 in the post-Ebola period as one new facility was added in the post-Ebola period. ^4^ Similarly, during the post-Ebola period, 5 MCHPs were upgraded to CHPs increasing the staffing requirement for the CHPs from 288 to 318.

By March 2017 and well into the post-Ebola period, a total of 1,389 health worker positions (medical and non-medical) were recommended by BPEHS, but only 371 (27%) were filled, resulting in an overall human resource deficit of 1,018 (73%).

## Discussion

This is the first study assessing staffing levels (medical and non-medical) 16 months into the post-Ebola period and comparing the status with pre- and intra-Ebola periods. The situation remains alarming with a 60% deficit for medical and 92% deficit for non-medical staff. We need to reiterate our earlier urgent call for bold policies and donor support that goes beyond “business as usual.” ^5^ In addition to enhancing staff training, further action could include rapid mobilization of financial resources for employment of non-medical and support staff, including those currently out of public service and reinstatement of retired medical personnel still fit enough to work ^2^. Importantly the macro-economic restrictions on the wage bill imposed by the International Monetary Fund (IMF) hamper recruitment and adequate salary levels ^4^. These need to be boldly tackled. Whether or not the BPEHS standards are realistic and adaptation thereof may also need consideration.

The strengths of the study are that we included all district public health facilities, all human resource cadres and similar data prior to, during and after the outbreak. The main limitation is that we might have excluded some staff not on regular payrolls (those working on a volunteer basis), although we believe this is unlikely to offset or negate our study findings.

There are two key messages from this study. First, at the current rate of 5% improvement in the medical staff deficit over the 16-month post-Ebola period (65% intra-Ebola to 60% post-Ebola), it will take an additional 12 years to achieve BPEHS standards - too little, too slow! 

Second, the persistent 92% gap for non-medical staff has major implications for future Ebola and infectious disease outbreaks ^6^. Essential services for infection prevention and control at health facilities and the implementation of personal hygiene measures and effective waste management depend on non-medical staff. In the unfortunate event of a new Ebola outbreak, the current scenario would result in a *déjà vu* of high transmission among health workers and the community at large ^7^. Ending the restrictive wage bill ^4^ is vital to mobilize the needed financial resources and rapidly employ and deploy staff.

In conclusion, with an overall health worker deficit of 1,018, 16 months into the post-Ebola period compared to a deficit of 1,026 during the Ebola outbreak, “nothing has really changed.” We reiterate our call for strong political will, international collaboration, generous funding and a change in hiring restrictions imposed by the IMF.

## Data availability

### Underlying data

Open Science Framework: Squire J. Squire_James_SORTIT2_HRH_data 2019. https://doi.org/10.17605/OSF.IO/QK5YG ^8^.

Data are available under the terms of the Creative Commons Zero “No rights reserved” data waiver (CC0 1.0 Public domain dedication).

The Sierra Leone Health Management Information Systems, the District Health Information System 2 (DHIS2), is accessible with a Ministry of Health and Sanitation login through https://sl.dhis2.org/. The Directorate of Policy, Planning, and Information (DPPI) can be contacted through Dr. Francis Smart ( drfsmart@gmail.com), Director, DPPI, MOHS, with an information request detailing the specific data request and purpose of use. Applicants will be asked to provide details of the reason for the request and details pertaining data request (such as data points, disaggregation, time period). In this case, data access would be granted to persons who request data for research purposes if they can provide appropriate ethical approval documentation.

## References

[1] MoHS: The Basic Package of Essential Health Services, 2015-2020.Freetown, Sierra Leone: Ministry of Health and Sanitation.2015 Accessed 14th January, 2016. Reference Source

[2] Sylvester Squire J Hann K Denisiuk O: The Ebola outbreak and staffing in public health facilities in rural Sierra Leone: who is left to do the job? *Public Health Action.* 2017;7(Suppl 1):S47–S54. 10.5588/pha.16.0089 28744439 PMC5515564

[3] Govindaraj R Herbst CH Clark JP: Strengthening Post-Ebola Health Systems: From Response to Resilience in Guinea, Liberia, and Sierra Leone. Washington, DC;2018 Reference Source

[4] Marphatia A Moussié R Ainger A: Confronting the contradictions: The IMF, wage bill caps and the case for teachers. Action AID,2007 Accessed 22 February 2019. Reference Source

[5] Philips M Markham A: Ebola: a failure of international collective action. *Lancet.* 2014;384(9949):1181. 10.1016/S0140-6736(14)61606-8 25218774

[6] Harries AD Zachariah R Tayler-Smith K: Keeping health facilities safe: one way of strengthening the interaction between disease-specific programmes and health systems. *Trop Med Int Health.* 2010;15(12):1407–12. 10.1111/j.1365-3156.2010.02662.x 21137105

[7] Dallatomasina S Crestani R Sylvester Squire J: Ebola outbreak in rural West Africa: epidemiology, clinical features and outcomes. *Trop Med Int Health.* 2015;20(4):448–54. 10.1111/tmi.12454 25565430 PMC4375518

[8] Squire J: Squire_James_SORTIT2_HRH_data. osf.io/qk5yg.2019 10.17605/OSF.IO/QK5YG

