# Supplementary material for: Staffing in public health facilities after the Ebola outbreak in rural Sierra Leone: How much has changed?
Source: F1000Res. 2020 Jan 9;8:793. Originally published 2019 Jun 6. [Version 2] doi: 10.12688/f1000research.18566.2 (PMC7047906; doi:10.12688/f1000research.18566.2)
Supplement: Peer review history

# Peer review: Reviewer response for version 2

**DOI:** 10.5256/f1000research.23802.r58463

**Published:** 2020-01-31

**Authors:** Armand Sprecher

**Affiliations:** 1 Médecins Sans Frontières, Brussels, Belgium

This paper has not undergone many changes from the original version.

 It has some added material in the introduction regarding the immediate post-Ebola period as an opportunity to fix the human resource deficit.

 The methods section has some material describing the healthcare human resources development pathway and states that the major obstacle is insufficient financial resources largely due to restriction imposed by the IMF. The cited reference, which, in fairness, did appear in the previous version as well, does support this, though it dates from 2007, and so the reader must assume that the current fiscal policy environment has not changed. As the paper's major recommendation is a reversal of IMF policy, some confirmation that this is still the major obstacle to Sierra Leone's healthcare human resources meeting the country's goals would be welcome if such a reference is available.

 The paper now has a sentence directing readers to the authors' previous work for more information on the details of the staffing shortfalls. A quick examination of the two tables mentioned does prove interesting. For example, the 92% of the 92% non-medical staff shortfall comes from just the cleaners and security officers. As the authors mention the consequences of insufficient waste management staff, and it would help the reader to have presented at least the quantification of the shortfall for this category to support their argument in this paper, as it would seem quite relevant.

I confirm that I have read this submission and believe that I have an appropriate level of expertise to confirm that it is of an acceptable scientific standard.

# Peer review: Reviewer response for version 2

**DOI:** 10.5256/f1000research.23802.r58464

**Published:** 2020-01-09

**Authors:** Hayk Davtyan

**Affiliations:** 1 TB Research and Prevention Center, Yerevan, Armenia

I confirm that I have read this submission and believe that I have an appropriate level of expertise to confirm that it is of an acceptable scientific standard.

# Peer review: Reviewer response for version 1

**DOI:** 10.5256/f1000research.20323.r53202

**Published:** 2019-09-17

**Authors:** Armand Sprecher

**Affiliations:** 1 Médecins Sans Frontières, Brussels, Belgium

This study gives a simple, but clear, description of the gap between Sierra Leone's health structure staffing ambitions and the actual state of affairs in Kailahun over the last several years. By merely measuring the difference between what the ministry of health and sanitation has set as staffing objectives and what it has achieved, the study is no more complicated than it needs to be. As such, the methods and results sections are suitably short and to the point.

 That being said, the paper would profit from going into greater depth in the discussion. This is hardly the first study to examine staffing shortfalls in Sierra Leone. The authors cite their own previous work, but there are other papers that merit mention in this domain. A quick PubMed search of "Sierra Leone Staffing" brings up a number of studies, some of which the authors may wish to include in their discussion of health structure staffing in Sierra Leone.

 The reader is also left in the dark as to how the Basic Package of Essential Health Services was put together and whether the levels set in this standard were tailored to well assessed needs in Sierra Leone, imported from another context, or are aspirational. As such, it is difficult for the reader to guess at the impact of the measured gap.

 The paper would benefit from some additional exploration of not meeting this standard. The authors speculate about the consequences of the severe non-medical under-staffing in terms of being unable to cope with infectious disease outbreaks because of insufficient hygiene staff, but at a 92% deficit of "administrative staff, cleaners, cooks, maintenance workers, drivers, and security personnel" (and this list should perhaps appear somewhere in the body of the paper and not just in a footnote to table 2), the day to day consequences should go well beyond this.

 The reader might also profit from some information about why the gap has persisted. The recommendation to mobilize additional financial resources suggests that the underlying problem is insufficient funding, which seems reasonable, but the foundation for this recommendation is never laid by any mention of what has caused the gap to remain. Perhaps the money is available but the qualified people are in the middle of their training programs? Other problems could be at work as well, but the authors do not explore this next logical step following from their findings.

 This is a nice simple study, but the readers would profit from the authors shedding more light on the meaning of their findings.

I confirm that I have read this submission and believe that I have an appropriate level of expertise to confirm that it is of an acceptable scientific standard, however I have significant reservations, as outlined above.

# Response

**Published:** 2019-12-03

**Authors:** Katrina Hann

**Affiliations:** Sustainable Health Systems, Sierra Leone

**Dear Armand Sprecher,**

**Thanks for reviewing the manuscript and your insightful comments. We have provided responses to each of your comments below. Your comments are highlighted in bold and our responses follow using normal font.**

**Reviewer comment 1. That being said, the paper would profit from going into greater depth in the discussion. This is hardly the first study to examine staffing shortfalls in Sierra Leone. The authors cite their own previous work, but there are other papers that merit mention in this domain. A quick PubMed search of "Sierra Leone Staffing" brings up a number of studies, some of which the authors may wish to include in their discussion of health structure staffing in Sierra Leone.**

- Response: Thanks for the comment. We do agree that other studies have looked at staffing issues in Sierra Leone. However, this is the first study that has assessed the implementation of the BPEHS in relation to the Ebola outbreak and the references we have included in the paper relate to this theme. We thank you for your understanding. 

**Reviewer comment 2: The reader is also left in the dark as to how the Basic Package of Essential Health Services was put together and whether he levels set in this standard were tailored to well assessed needs in Sierra Leone, imported from another context, or are aspirational. As such, it is difficult for the reader to guess at the impact of the measured gap.**

- Response: The BPEHS is the national standard that stipulates the minimum staffing for each type of public health facility level, which is critical to maintain service delivery standards. The BPEHS has been described before and we have included its details and justification for its use in the introduction. The standards are not aspirational but a necessary requirement.   

**Reviewer comment 3:  The paper would benefit from some additional exploration of not meeting this standard. The authors speculate about the consequences of the severe non-medical under-staffing in terms of being unable to cope with infectious disease outbreaks because of insufficient hygiene staff, but at a 92% deficit of "administrative staff, cleaners, cooks, maintenance workers, drivers, and security personnel" (and this list should perhaps appear somewhere in the body of the paper and not just in a footnote to table 2), the day to day consequences should go well beyond this.**

- Response. Thank you for this important comment. In the related article that was already published and duly referenced entitled “The Ebola outbreak and staffing in public health facilities in rural Sierra Leone: who is left to do the job?”, we disaggregated the staffing deficit by cadre. We cited this article in the current study. Since there were no significant changes in the staffing levels, we decided not to duplicate the same result in the current study. We have included a reference to the full list of medical and non-medical staff.

**Reviewer comment 4. The reader might also profit from some information about why the gap has persisted. The recommendation to mobilize additional financial resources suggests that the underlying problem is insufficient funding, which seems reasonable, but the foundation for this recommendation is never laid by any mention of what has caused the gap to remain. Perhaps the money is available but the qualified people are in the middle of their training programs? Other problems could be at work as well, but the authors do not explore this next logical step following from their findings.**

- Response: In the discussion we have mentioned the possible reasons (which are multiple) and actions that needs to be taken to address some of the root causes and why corrective action has not been forthcoming. In future research, we hope to consider determining the root causes

**Reviewer comment 5: This is a nice simple study, but the readers would profit from e authors shedding more light on the meaning of their findings.**

- Response: In the related article that was already published and duly referenced entitled “The Ebola outbreak and staffing in public health facilities in rural Sierra Leone: who is left to do the job?” we already touched the issues around IMF restrictions and retention issues and tried to avoid duplication in this current paper. We thank you for your kind consideration.  

# Peer review: Reviewer response for version 1

**DOI:** 10.5256/f1000research.20323.r53419

**Published:** 2019-09-11

**Authors:** Hayk Davtyan

**Affiliations:** 1 TB Research and Prevention Center, Yerevan, Armenia

The article is very well written with clearly defined aim/research question and methodology designed to answer it.

 The question of interest is of high importance as it relates to Ebola (highly contagious and dangerous virus) which could become a threat for the whole world. The problem discussed in the article (under-staffing) is worrisome and calls for an urgent actions.

 It seems there is a need for more information on settings. Particularly regarding financing and hiring/staffing of the health facilities in the article. 

 While the presentation of the problem is clear it will be really interesting and important to understand root-causes of the problems which may help to solve the issues discussed. Most importantly if there was a standard setting for minimum staff levels by the Ministry (so, assuming there is political will) then what are the constrains for not increasing number of human resources. The lack of qualified professionals may cause the under-staffing of medical resources but we observe no change in the number of non-medical staff so there should be other causes preventing the capacity increase in terms of human resources. If the data is not available researchers might want to address it in the limitations and call for further investigation of the topic.

I confirm that I have read this submission and believe that I have an appropriate level of expertise to confirm that it is of an acceptable scientific standard, however I have significant reservations, as outlined above.

# Response

**Published:** 2019-12-03

**Authors:** Katrina Hann

**Affiliations:** Sustainable Health Systems, Sierra Leone

**Dear Hayk Davtyan**

**Many thanks for reviewing the manuscript and your insightful comments and contributions. We have provided responses to each of your comments below. The review comments are in bold font and we have responded using bullet points and normal font.**

**Reviewer comment 1: It seems there is a need for more information on settings. Particularly regarding financing and hiring/staffing of the health facilities in the article.**

 

- Response: Thank you for this important comment. As suggested, we have included more information on the settings specifically addressing your concerns.

 

**Reviewer comment 2.** **While the presentation of the problem is clear it will be really interesting and important to understand root-causes of the problems which may help to solve the issues discussed.**

 

- Response: The current study’s aim and objectives were to identify what is the deficit (the gap) in the health workforce in relation to Basic stipulated standards and not to address root-causes of the deficit.

- In the discussion we mentioned the possible reasons and actions that needs to be taken to address some of the root causes. In future research, we hope to consider determining the root causes.

**Reviewer comment 3: Most importantly if there was a standard setting for minimum staff levels by the Ministry (so, assuming there is political will) then what are the constrains for not increasing number of human resources.**

 

- Response: The BPEHS is the national standard that stipulates the minimum staffing for each type of public health facility, which is critical to maintain service delivery standards. The BPEHS target is to be achieved by 2020. However, with the current pace of implementation, this target will not be achieved by 2020. The constraints in increasing the health workforce are multiple and include limited available funds, availability of trained manpower, IMF restrictions etc. We have already highlighted the constraints and the urgent measures that need to be implemented to address the underlying staffing deficits.

 

**Reviewer comment 4: The lack of qualified professionals may cause the under-staffing of medical resources but we observe no change in the number of non-medical staff so there should be other causes preventing the capacity increase in terms of human resources. If the data is not available researchers might want to address it in the limitations and call for further investigation of the topic.**

Response: We have already discussed the underlying factors contributing to the observed deficits in both medical and non-medical staffing levels. We agree with the reviewer that more research is needed and have suggested areas that merit further research and investigation (in the Discussion).

# Peer review: Reviewer response for version 1

**DOI:** 10.5256/f1000research.20323.r53417

**Published:** 2019-09-04

**Authors:** Wendemagegn Enbiale

**Affiliations:** 1 Department of Dermatovenerology, College of Medicine and Health Sciences, Bahir Dar University, Bahir Dar, Ethiopia

I have found the study very interesting and partially relevant in flagging the deficit for minimal staffing by label of care (based on the BPEHS standards) in Sierra Leone. 

 Some of my question and concerns are:

1. Health Workforce (HWF) staffing usually measured based on population and there are intentional standards for developing countries. Why the authors are not interested to use or at least mention that?
2. What is the catchment population of those health facilities and what is the health workforce staffing deficit based on the WHO criteria (for a developing country)?
3. Do you need the HWF just to fulfil the BPEHS standard or is there any evidence suggesting compromisation of the routine health care service because of the stated HWF deficit. 
4. For prioritization and focused action, I would recommend the showing the dis-aggregated deficit from nurses, midwifes and doctors which would be much more important for the policy maker for action.

I confirm that I have read this submission and believe that I have an appropriate level of expertise to confirm that it is of an acceptable scientific standard, however I have significant reservations, as outlined above.

# Response

**Published:** 2019-12-03

**Authors:** Katrina Hann

**Affiliations:** Sustainable Health Systems, Sierra Leone

Dear Dr Wendemagegn Enbiale, Thank you for your review and insightful comments on the manuscript. We have responded to each of your points below. 1. Reviewer comment 1. Health Workforce (HWF) staffing usually measured based on population and there are international standards for developing countries. Why the authors are not interested to use or at least mention that? Response: Thank you for this comment. The Basic Package for Essential Health Services (BPEHS) is the national standard used to guide health workforce requirements in Sierra Leone. Despite the existence of global methods usually using a population-based measure the BPEHS has been adapted to the Sierra Leone content and thus more appropriate to measure health workforce gaps. In particular, the BPEHS allows us to quantify the real gap between the Government’s commitment and the actual status in terms of staffing. In addition, the international standard for staffing measures focus only on clinical staff. However, in the Ebola and post-Ebola context, non-clinical staffing are equally essential, especially in terms of infection prevention and control measures. Therefore, the use of the BPEHS is more appropriate as it allows assessment of both medical and non-medical staff cadres. In the introduction, we have added more explanation and references to clarify our stand to use the BPEHS as the yardstick in the introduction. We have also added more information of the BPEHS standard in the methods section. 2. Reviewer Comment 2: What is the catchment population of those health facilities and what is the health workforce staffing deficit based on the WHO criteria (for a developing country)? Response: This study is focused on a specific district, Kailahun and all health facilities in the district was included. Details of the Study setting and population was already reported in the previous paper for which this study is a follow up. We have referenced this paper and its contents in case the reader might wish for more information. As justified in point 1 above, since we utilised the BPEHS national standards, we do not see applying the WHO criteria as relevant to this study. 3. Reviewer comment 3: Do you need the HWF just to fulfil the BPEHS standard or is there any evidence suggesting compromiation of the routine health care service because of the stated HWF deficit. Response: Ensuring basic standards of Human resources for health, is one of the WHO health systems building blocks. It is essential to achieve this requirement if health facilities are to have the “hands on deck” to ensure proper and effective functioning of health systems to support the delivery of care to the population. The 2014-2016 Ebola outbreak with the resulting decline in the health workforce resulted in the collapse of even the most basic of routine health services. The BPEHS is the national standard that stipulate the minimum staffing for each type of public health facility, which is critical to maintain service delivery standards and our study shows that we are far from these standards. This gap will need to be filled. We have now added references to support the evidence on the health workforce deficit and its detrimental effect on health service delivery. 4. Reviewer comment 4: For prioritization and focused action, I would recommend the showing the dis-aggregated deficit from nurses, midwifes and doctors which would be much more important for the policy maker for action. Response: Thank you for this important comment. In the already published article “The Ebola outbreak and staffing in public health facilities in rural Sierra Leone: who is left to do the job?” which has been referenced in the current study, we disaggregated the staffing deficit by cadre. Since there were no significant changes in the staffing levels, we decided not to duplicate the same results in the current study. We thank you for your kind understanding.
